# Combination of aloe emodin, emodin, and rhein from Aloe with EDTA sensitizes the resistant *Acinetobacter baumannii* to polymyxins

**DOI:** 10.3389/fcimb.2024.1467607

**Published:** 2024-09-13

**Authors:** Yue Zhao, Tingting Zhang, Yinping Liang, Xiaoqing Xie, Hongwei Pan, Meng Cao, Shuhua Wang, Dalei Wu, Jing Wang, Chuandong Wang, Wei Hu

**Affiliations:** ^1^ College of Pharmacy, Shandong University of Traditional Chinese Medicine, Jinan, China; ^2^ State Key Laboratory of Microbial Technology, Microbial Technology Institute, Shandong University, Qingdao, China; ^3^ Department of Clinical Laboratory, Qilu Hospital of Shandong University, Jinan, Shandong, China; ^4^ Research and Development Center, Shandong Aobo Biotechnology Co., Ltd, Liaocheng, Shandong, China

**Keywords:** *Acinetobacter baumannii*, polymyxins resistance, Aloe, anthraquinone components, ethylenediaminetetraacetic acid

## Abstract

**Background:**

The continuous emergence and spread of polymyxin-resistant *Acinetobacter baumannii* pose a significant global health challenge, necessitating the development of novel therapeutic strategies. Aloe, with its long-standing history of medicinal use, has recently been the subject of substantial research for its efficacy against pathogenic infections.

**Methods:**

This study investigates the potential application of anthraquinone components in aloe against polymyxin-resistant *A. baumannii* by liquid chromatography-mass spectrometry, in vitro activity assessment, and construction of animal infection models.

**Results:**

The findings demonstrate that aloe emodin, emodin, rhein, and their mixtures in equal mass ratios (EAR) exhibit strain-specific antibacterial activities against polymyxin-resistant *A. baumannii*. Co-administration of EAR with EDTA synergistically and universally enhanced the antibacterial activity and bactericidal efficacy of polymyxins against polymyxin-resistant *A. baumannii*, while also reducing the frequency of polymyxin-resistant mutations in polymyxinssensitive *A. baumannii*. Following toxicity assessment on human hepatic and renal cell lines, the combination therapy was applied to skin wounds in mice infected with polymyxin-resistant *A. baumannii*. Compared to monotherapy, the combination therapy significantly accelerated wound healing and reduced bacterial burden.

**Conclusions:**

The combination of EAR and EDTA with polymyxins offers a novel therapeutic approach for managing skin infections caused by polymyxinresistant *A. baumannii*.

## 1 Introduction

*Acinetobacter baumannii* is one of the most common opportunistic pathogens responsible for nosocomial infections globally, particularly prevalent in intensive care unit (ICU) settings where it is a leading causative agent of ventilator-associated pneumonia (Vázquez-López et al., 2020). In addition to causing pneumonia, meningitis, and bacteremia, *A. baumannii* also represents a source of nosocomial skin and soft-tissue infections in the context of war wounds, surgical sites, and burns (Al Salman et al., 2020; Cavallo et al., 2023). Due to its ability to resist third-generation cephalosporins and carbapenems, the treatment of *A. baumannii* infections faces severe challenges, with a global average mortality rate as high as 42.6%, thereby posing a significant threat to public health (Mohd Sazlly Lim et al., 2019; Ikuta et al., 2022; Roch et al., 2023). According to data from the Centers for Disease Control and Prevention of the USA, the incidence of multidrug-resistant *A. baumannii* is four times higher than that of other Gram-negative pathogens such as *Klebsiella pneumoniae* and *Pseudomonas aeruginosa* (Giammanco et al., 2017). In response to this challenge, polymyxins have been reserved as “last-line” therapies in clinical practice for treating infections caused by multidrug-resistant or extensively drug-resistant *A. baumannii* (Nang et al., 2021; Shields et al., 2023). Polymyxins, such as polymyxin B (PMB) and colistin (CST), are lipopeptide antibiotics employed in clinical settings. Their antibacterial mechanism involves targeting lipid A, a constituent of the bacterial outer membrane’s lipopolysaccharide (LPS), leading to disruption of the outer membrane’s integrity (Ledger et al., 2022). However, due to its increased use in clinical practice, polymyxin-resistant strains have emerged worldwide (Lima et al., 2020; Dwibedy et al., 2024; Luo et al., 2024). Currently, *A. baumannii* demonstrates a multifaceted mechanism and diversified evolution of polymyxin resistance, including intrinsic mechanisms and acquired mechanisms mediated by plasmids (Wang et al., 2022; Shahzad et al., 2023; Liu et al., 2024).

In addition to the recommendations for more prudent dosing strategies and systematic monitoring for polymyxin-resistance (Pogue et al., 2017; Lima et al., 2020), the development of novel synthetic and semi-synthetic analogues of polymyxins, along with the concurrent use of polymyxins with antibiotics or non-antibiotic adjuvants, represents a preferred strategy for addressing polymyxins resistance (Koh Jing Jie et al., 2022; Ardebili et al., 2023; Slingerland and Martin, 2024). The strategy of combination therapy, owing to its economic and safety advantages, has emerged as a highly promising approach to overcoming antibiotic resistance (Yang et al., 2020). For example, ethylenediaminetetraacetic acid (EDTA), a common pharmaceutical excipient and chelating agent widely used in medicine and the food industry, has gained considerable attention in the antimicrobial field due to its strong metal ion chelating ability, leading to the development of various combination therapies (Khare et al., 2021; Chang et al., 2022; Bari et al., 2023). A study reported that the combination therapy of colistin and EDTA exhibited potent synergistic effects both *in vitro* and *in vivo* against *mgrB*-mediated colistin-resistant *Klebsiella pneumoniae*, offering a novel approach for treating extensively drug-resistant (XDR) bacterial infections (Shein et al., 2022). Furthermore, polymyxin B and EDTA showed synergistic inhibitory effects on *Pseudomonas aeruginosa* and *Staphylococcus aureus*, and were suggested for the management of biofilm-associated conditions, particularly those amenable to topical therapies such as cystic fibrosis (Hale et al., 2024).

The Earth harbors an estimated 250,000 to 500,000 plant species, each of which contains a diverse array of phytochemical compounds that represent a vast repository of potential therapeutics (Borris, 1996). Many plant-derived antimicrobials have been widely applied in the medical field, and their combination with antibiotics can reduce the required antibiotic concentrations and re-sensitize multidrug-resistant pathogens to these drugs (Song et al., 2021). It has been demonstrated that natural flavonoids with catechol-type structures, such as 7,8-dihydroxyflavone, myricetin, and luteolin, are able to enhance the bactericidal effect of colistin by disrupting the iron homeostasis of Enterobacteriaceae, providing a reliable strategy for potentiating colistin in clinical treatments (Zhong et al., 2023). Another study discovered that extracts from *Silene armeria*, when used in combination with polymyxin B, exhibited synergistic antibacterial activity against *A. baumannii.* The active compound 6-bromo-2-naphthol was identified as the primary synergist with polymyxin B, offering a new direction for effectively controlling multidrug-resistant *A. baumannii* (Kang et al., 2022). Thus, guided by the principles of green pharmacy, safe medication practices, and a return to natural remedies, research exploring natural compounds as potential solutions to the global antimicrobial resistance crisis has garnered growing interest and focus.

Aloe, belonging to the genus *Aloe* in the family Liliaceae, is a highly adaptable plant distributed worldwide. It exhibits various pharmacological properties, including immunomodulatory, anticancer, antibacterial, anti-inflammatory, and wound healing effects, endowing aloe with widespread applications in medicine, food, and cosmetics (Ushasree et al., 2024). Currently, reports on the antibacterial activity of aloe predominantly focus on Gram-positive bacteria (Ushasree et al., 2024), while antibacterial activity against Gram-negative bacteria, particularly polymyxin-resistant *A. baumannii*, remains scarce. Therefore, this study investigated four different varieties of aloe plants and their major active components, aiming to reveal the potential of aloe-derived natural compounds as an effective anti-infective strategy against polymyxin-resistant *A. baumannii*.

## 2 Materials and methods

### 2.1 *Acinetobacter baumannii* strains, Aloe and antimicrobial agents

The *A. baumannii* strains used in this study were listed in **Table 1**, among which the type strain ATCC 19606 and 17978 are polymyxin-susceptible, six clinical strains were isolated from Qilu Hospital of Shandong University and identified as polymyxin-resistant *A. baumannii* isolates, and 10213 was obtained by ATCC 19606. All *A. baumannii* strains were routinely grown at 37°C in tryptone soy broth (TSB, Beijing Solarbio Science & Technology Co., Ltd., China). Four different varieties of aloe plants leaves, *i.e.*, *Aloe arborescens* Miller (AA), *Aloe barbadensais* Miller (AB), *Aloe ferox* Miller (AF), and *Aloe vera* L. var. *chinensis* (Haw.) Berger (AL), were purchased from Jiangsu Shuyang Meiruoxiahua Co., Ltd., China. All antibiotics and compounds used in this study were obtained from Shanghai Yuanye Bio-Technology Co., Ltd. (Shanghai, China).

**Table 1:** The MIC and MBC of reagents against *A. baumannii* strains (mg/L).

Strains	Polymyxin B		Colistin		Emodin		Aloe Emodin		Rhein		Aloesin		EAR		ABCE		EDTA	
	MIC	MBC	MIC	MBC	MIC	MBC	MIC	MBC	MIC	MBC	MIC	MBC	MIC	MBC	MIC	MBC	MIC	MBC
19606	0.5	0.5	1	1	>512	>512	>512	>512	>512	>512	>512	>512	>512	>512	10000	>10000	>1024	>1024
17978	0.5	0.5	1	1	>512	>512	>512	>512	>512	>512	>512	>512	>512	>512	10000	>10000	>1024	>1024
10213	>512	>512	>512	>512	0.5	32	1	64	32	>256	>1024	>1024	2	64	312	10000	>1024	>1024
07AC366	64	64	256	256	>1024	>1024	>1024	>1024	>1024	>1024	>1024	>1024	4	>256	10000	>10000	>1024	>1024
189B	>512	>512	>512	>512	512	512	256	256	512	512	>1024	>1024	16	64	5000	>10000	>1024	>1024
1415E	64	64	>512	>512	>1024	>1024	>1024	>1024	>1024	>1024	>1024	>1024	>512	>512	>10000	>10000	>1024	>1024
6588E	256	256	512	512	0.5	512	2	4	128	128	>1024	>1024	2	4	312	5000	>1024	>1024
038E	>512	>512	512	512	0.5	>512	4	4	128	256	>1024	>1024	2	4	625	10000	>1024	>1024
245B	>512	>512	512	512	2	32	2	2	64	128	>1024	>1024	1	2	625	10000	>1024	>1024

EAR, emodin, aloe emodin and rhein were mixed in a mass ratio of 1:1:1; ABCE, crude extracts from *Aloe barbadensis*; EDTA, ethylenediaminetetraacetic acid; MIC, minimum inhibitory concentration; MBC, minimum bactericidal concentration.

### 2.2 Preparation of natural deep eutectic solvents

Choline chloride, D-(+)-glucose, malic acid, citric acid and lactic acid were used to prepare the natural deep eutectic solvents (NADESs). As previously reported (Dai et al., 2013; Wu et al., 2018), different NADESs were employed in this study with the molar ratios as choline chloride:D-glucose (1:1), choline chloride:malic acid (2:1), choline chloride:citric acid (2:1), and lactate:D-glucose (5:1), respectively. The mixtures were slowly heated in a water bath at a temperature of 80°C with continuous stirring for a period of 30 to 60 minutes until a uniform, transparent, and clear liquid was formed.

### 2.3 Compounds extraction and purification

The fresh leaves of aloe were washed with water, cut into small pieces and homogenized for crushing. The resulting mixture was then filtered through a clean muslin cloth to remove any residual plant matter, and the supernatant was collected. Subsequently, the liquid extract was then pre-frozen at -80°C for 12 h, followed by freeze-drying using a Crystal Alpha freeze dryer (Germany). The freeze-drying process consisted of a 40-minute freezing step, an 8-hour primary drying step, and a 4-hour secondary drying step, yielding a fine powder. Ultrasound-assisted extraction was performed in a 20 mL round-bottom flask with 0.25 g of aloe powder and 10 mL of NADES solvent. The mixture was subjected to ultrasonication (KQ5200B, 200 W, 40 kHz, KunShan, China) at 80°C for 30 min. After extraction was completed, the solutions were collected and centrifuged at 10000 rpm/min for 15 min. All crude extracts used in the experiments were stored at -20°C under light-protected conditions. The extracts used in subsequent experiments were all taken from the same batch of samples to minimize the impact of batch-to-batch variation on the experimental results. The supernatant was diluted 100-fold with methanol and then filtered using a 0.22 µm organic filter membrane for subsequent HPLC injection. After being concentrated by rotary evaporation, the crude extracts were dissolved in methanol and subsequently subjected to separation using Sephadex LH-20 gel column chromatography. Methanol was used for elution at a controlled flow rate of 30 s per drop under atmospheric pressure, with fractions collected at 10-mL intervals. Based on the colorimetric results of thin-layer chromatography, the fractions were combined and then concentrated by evaporation. Compounds in the fractions were further separated by semipreparative high performance liquid chromatography (HPLC, ThermoFisher) equipped with a 250 × 10 mm Luna C_18_ column (Phenomenex) eluting with gradient eluent of methanol in water from 60% to 100% at 30°C at a flow rate of 1.8 mL/min with 254 nm UV spectrum.

### 2.4 HPLC-MS analysis

HPLC analysis was performed with a Thermo Scientific Ultimate 3000 HPLC system using a column (Thermo Fisher Scientific, C_18_, 4.6 × 250 mm, 5 μm) equipped with a guard column. The binary mobile phase consisted of water (A) and methanol (B) in a linear gradient program from 5% B to 100% B in 46 min at a flow rate of 0.8 mL/min. The methanol concentrations were changed as follows: 5 ~ 100% B (0 ~ 30 min), 100% B (30 ~ 40 min), 5% B (40 ~ 46 min). The column temperature was as 30 °C and the sample volume injected was 10 μL. Coupled MS analysis was performed on an Impact HD Q-TOF (Bruker, Germany) equipped with an ESI source. Diode array detector spectra were acquired at 254 nm over a scan range of 190 ~ 400 nm. ESI source parameters were used as follows: negative ion mode, heater temperature 200°C, nitrogen (purity ≥ 99.99%) pumped into the ion source at a rate of 4 L/min, sheath gas flow rate 50 arb, spray voltage 3.5 kV, and scanning ranges of *m/z* 50 ~ 1500 for both MS and MS/MS mass spectra.

### 2.5 Zone of inhibition assay

Broth cultures of *A. baumannii* COLR 10213 in the logarithmic growth phase were collected and resuspended to 1.0×10^8^ CFUs/mL. A sterile cotton swab was soaked in *A. baumannii* COLR 10213 culture until fully absorbed. Then the surface of the TSB plate was coated evenly by the sterile cotton. Subsequently, 100 μL aloe liquid extracts (25 mg/mL) was added in the sterile Oxford cup and placed on the TSB agar plate. The same volume of solvent or solution containing 10 mg/L CST were used as controls. The plates were gently placed into an incubator at 37°C for 24 h until the clear inhibition zone were observed and their diameters were recorded.

### 2.6 Antibacterial and bactericidal activity analysis

Minimum inhibitory concentrations (MICs) of all compounds were determined by the broth microdilution assay according to the Clinical and Laboratory Standards Institute (CLSI) recommendations for *A. baumannii* (CLSI, 2021). Briefly, drugs or crude extracts were 2-fold diluted in Mueller-Hinton broth (Oxoid, UK) mixed with bacterial suspensions containing approximately 5 × 10^5^ colony forming units (CFUs)/mL in a sterilized 96-well microliter plate (NEST Biotechnology, China). After 18 ~ 24 h incubation at 37°C, the MIC values were defined as the lowest concentrations of drugs with no visible growth of bacteria. After the MIC determination, 50 μL aliquots from all the wells which showed no visible bacterial growth were seeded on TSB agar plates and incubated at 37°C for 24 h. Minimum bactericidal concentration (MBC) was recorded as the lowest concentration killing 99.9% of the bacterial population (CLSI, 2019).

### 2.7 Checkerboard assay

A microdilution checkerboard method was used to determine the potential effects of individual compound combinations (Ju et al., 2022). The interactions were evaluated using the fractional inhibitory concentration index (FICI). The FICI was defined as (MIC_A in combination/_MIC_A alone_) + (MIC_B in combination/_MIC_B alone_). The interaction inferred from the resulting FICI values was assessed according to the following criteria: synergy, ≤ 0.5; additivity, > 0.5 to ≤ 1; indifference (no interaction), > 1 to ≤ 4; antagonism, > 4.

### 2.8 Time-kill curve measurement

Bacterial cultures in the exponential phase were diluted into MHB media to a final concentration of 5 × 10^5^ CFUs/mL. The bacterial suspensions were supplemented with varying concentrations of the compounds, either alone or in combination. The mixture was incubated at 37°C for 0, 2, 4, 6, 12, and 24 h, respectively. Following a 10-fold serial dilution on MHB plates, the number of surviving bacterial colonies was counted after overnight incubation.

### 2.9 Determination of the frequency of polymyxin-resistant mutation

*A. baumannii* 19606 and 17978 were cultured in TSB overnight at 37°C. After adjusting the concentration of bacteria suspensions to 1 × 10^9^ CFUs/mL, 300 μL of 1 × 10^9^ CFUs/mL were added to TSB plates containing 10 mg/L PMB or CST supplemented with or without varying concentrations of EAR and 256 mg/L EDTA. The TSB plates were incubated at 37°C for 24 h and the number of colonies grown was recorded.

### 2.10 Cytotoxicity assays

To determine the cytotoxicity of reagents, the human hepatic (HEP3B) and renal (HEK293) cells were maintained in Dulbecco’s Modified Eagle Medium supplemented with 10% fetal bovine serum (NEST Biotechnology, Wuxi, China) and 1% penicillin/streptomycin (Beijing Solarbio Science & Technology Co., Ltd.). A 250 µL of cell culture solution with a cell density of 5 × 10^4^ cells/mL was transferred to each well of a 48-well plate and incubated at 37°C in 5% CO_2_ atmosphere for 24 ~ 48 h. Test compounds at various concentrations (256 mg/L EDTA, 0 ~ 128 mg/L EAR, and 0 ~ 16 mg/L PMB or CST) with incubation medium were supplemented and cultivated for additional 24 h in the same conditions. Finally, 25 μL of the CCK-8 Cell Proliferation and Cytotoxicity Assay Kit (Beijing Solarbio Science & Technology Co., Ltd.) was added into each well, and OD at 450 nm was measured using a multifunction microplate reader (Infinite M200 Pro, Tecan) after incubation for 1 h at 37°C. The cell suspension supplemented with CCK-8 reagent was used as the control group, while the culture medium supplemented with CCK-8 reagent served as the black group. Cell viability was calculated using the following formula: cell viability (%) = (A_sample_ - A_blank_)/(A_control_ - A_blank_) × 100, where A presented absorbance at 450 nm.

### 2.11 Mice skin wound model

A mice skin wound model of polymyxin-resistant *A. baumannii* infection was developed as previously reported with some modification (Wang et al., 2024). Briefly, after removing the dorsal hair of mice and sterilizing with cotton swabs dipped in 75% ethanol solution, a sterile disposable biopsy punch (Rapid Core, Ted Pella, Inc., USA) was used to produce a full-thickness excision wound of 11-mm in diameter on each BALB/c mouse (Jinan Pengyue Experimental Animal. Breeding Co., Ltd, China). A sterile cotton swab was soaked in *A. baumannii* 1415E culture (1 × 10^8^ CFUs/mL) until fully absorbed. The bacterial culture was gently applied to the mouse skin wound using a wet swab and allowed to air dry at room temperature, with this procedure repeated for three consecutive days to establish continuous infection. PBS buffer was used as negative control. On the 5^th^ and 6^th^ d after the infection, the mice were euthanized by cervical dislocation. The wound cavity was collected and homogenized, followed by dilution with sterile saline and spreading onto agar plates. The plates were subsequently incubated at 37°C for 24 h to facilitate bacterial growth, enabling the determination of bacterial load through colony counting.

### 2.12 Treatment of the infected mice

After successful establishment of the infection by *A. baumannii* 1415E, mice were randomly assigned to groups with each group consisting of six mice. Subsequently, the wound sites were gently swabbed daily using cotton swabs soaked in a solution containing 32 mg/mL EDTA, 4 mg/mL EAR, and 2 mg/mL PMB. The PBS solution was utilized as the blank control group, whereas 2 mg/mL PMB was employed as the comparative group. The progress of wound healing in mice was observed daily, alongside measurements of wound diameter to calculate wound area. On the 12^th^ d of treatment, the bacterial load in wound cavity was detected as described above.

### 2.13 Statistical analysis

Each measurement was performed with a minimum of three replicates. The data are expressed as Mean ± SD (standard deviation). *P*-values were calculated using Student’s t-test for comparisons between two groups, the significance levels were denoted as follows: *, *P* < 0.05, **, *P* < 0.01, ***, *P* < 0.001. NS, not significant. Unless specified otherwise, the comparisons were made against the control group. The statistical analysis was performed using GraphPad Prism 9.5 software.

## 3 Results

### 3.1 Aloe emodin, emodin, and rhein in aloe extracts exhibit strain-specific antibacterial and bactericidal activities against polymyxin-resistant *A. baumannii*

**Figure 1:**
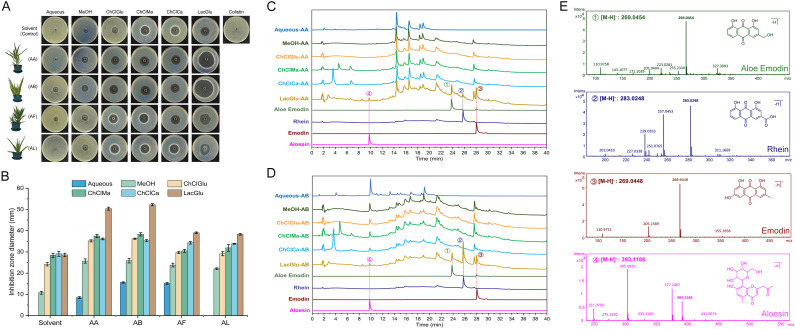
Activities of crude extracts from various *Aloe* species against *A. baumannii* COLR 10213 and component analysis of extracts from *A. arborescens* and *A. barbadensis*. **(A)** Representative inhibition zones against polymyxin-resistant *A. baumannii* COLR 10213 produced by the crude extracts from various *Aloe* species obtained using different solvent systems. The first panel shows the results of the respective solvent and 10 mg/L CST solution as controls. **(B)** Diameters of inhibition zones (mm) produced by the crude extracts from aloes in triplicate experiments (mean ± standard deviation, n = 3). **(C)** HPLC fingerprint chromatograms of crude extracts from *A. arborescens* (AA) using different solvent systems, with peak alignment at 254 nm wavelength. Chromatographic peaks of aloe emodin, rhein, emodin, and aloesin serve as standards for comparing retention times. **(D)** HPLC fingerprint chromatograms of crude extracts from *A. barbadensis* (AB). **(E)** High-resolution mass spectrometry analysis of indicated peaks (denoted as peaks 1-4 in panel **C, D**) in crude extracts of AA and AB using the Lac-Glu solvent. AA, *A. arborescens*; AB, *A. barbadensis*; AF, *A. ferox*; AL, *A. vera* L. var. *chinensis* (Haw.) Berger; MeOH, methanol; ChClGlu, choline chloride/D-glucose in a molar ratio of 1:1; ChClMa, choline chloride/malic acid in a molar ratio of 2:1; ChClCa, choline chloride/citric acid in a molar ratio of 2:1; LacGlu, lactic acid/D-glucose in a molar ratio of 5:1.

The fresh leaves of four different varieties of aloe plants (AA, AB, AF, and AL) were extracted using water (Aqueous), methanol (MeOH), or NADESs (ChCl-Glu, ChCl-Ma, ChCl-Ca, and Lac-Glu). The crude extracts were assessed for their antimicrobial activity using ZOI assay, employing the high-level polymyxin-resistant *A. baumannii* COLR10213 as the indictive strain. As shown in **Figures 1A, B**, the inhibition zones generated by NADES extracts were larger than the water or alcohol extracts, among which the inhibition by Lac-Glu extracts was the most pronounced. The extracts by Lac-Glu from AA and AB exhibited a larger zone against COLR10213 (with diameters of 50.04 ± 0.7 mm and 52.2 ± 0.5 mm, respectively), compared to those from AF and AL (with diameters of 39.0 ± 0.4 mm and 38.3 ± 0.4 mm, respectively). Next, the major components in crude extracts from AA and AB by NADESs were analyzed using HPLC. As shown in **Figures 1C, D** and **Supplementary Figure 1**, the midsection distribution of chromatographic peaks for different NADES extracts was generally consistent. Compared with other NADES extracts, Lac-Glu extract of AA (LacGlu-AA) and AB (LacGlu-AB) exhibited three prominently different peaks with relatively high contents between retention times of 24 and 28 minutes in the latter half, respectively denoted as peaks 1 to 3. A distinct differential peak was detected in the relatively polar front half with a retention time of approximately 10 minutes, denoted as peak 4. According the tested HPLC retention time (**Supplementary Figure 2**) of specific chemical components in aloe (Liang et al., 2021), peaks 1-4 were preliminarily identified aloe emodin, rhein, emodin, and aloesin, respectively. Through high-resolution MS (**Figure 1E**) and MS/MS (**Supplementary Figure 3**) analysis, these identifications were further confirmed. Peaks 1-4 were subsequently purified from the crude extracts following protocols described in the method, and compounds were further confirmed by comparing their retention times and ultraviolet absorption spectroscopy against standard aloe emodin, aloesin, emodin, and rhein (**Supplementary Figure 4**).

The antibacterial and bactericidal activities of the isolated compounds were analyzed against seven polymyxin-resistant *A. baumannii* strains, including 10213 (**Table 1**). Among the four compounds, aloe emodin, emodin, and rhein showed pronounced antibacterial (MIC ranged in 0.5 ~ 2 mg/L) and bactericidal (MBC 2 ~ > 1024 mg/L) activities compared to aloesin (MIC > 1024 mg/L, MBC > 1024 mg/L). Aloe emodin, emodin, and rhein demonstrated higher antibacterial activity (MIC 0.5 ~ 32 mg/L) against *A. baumannii* 10213, 6588E, 038E, and 245B but lower antibacterial activity (MIC 256 ~ 1024 mg/L) against *A. baumannii* 07AC366, 189B, and 1415E. Considering the potential synergy of active ingredients in medicinal plants (Williamson, 2001; Hu et al., 2023), a mixture named as EAR was prepared by combining equal mass ratios of aloe emodin, emodin, and rhein. In addition to maintaining the high antibacterial and bactericidal activities against *A. baumannii* 10213, 6588E, 038E, and 245B (MIC 1 ~ 2 mg/L, MBC 2 ~ 64 mg/L), EAR notably enhanced its effectiveness against *A. baumannii* 07AC366 and 189B (MIC 4 ~ 16 mg/L, MBC 2 ~ >256 mg/L). However, EAR did not exhibit any significant activity against *A. baumannii* 1415E (**Table 1**). These findings highlighted the potential variability in EAR’s efficacy across different polymyxin-resistant *A. baumannii* strains.

### 3.2 Combination treatment synergistically restored the sensitivity of resistant *A. baumannii* strains to polymyxins

**Figure 2:**
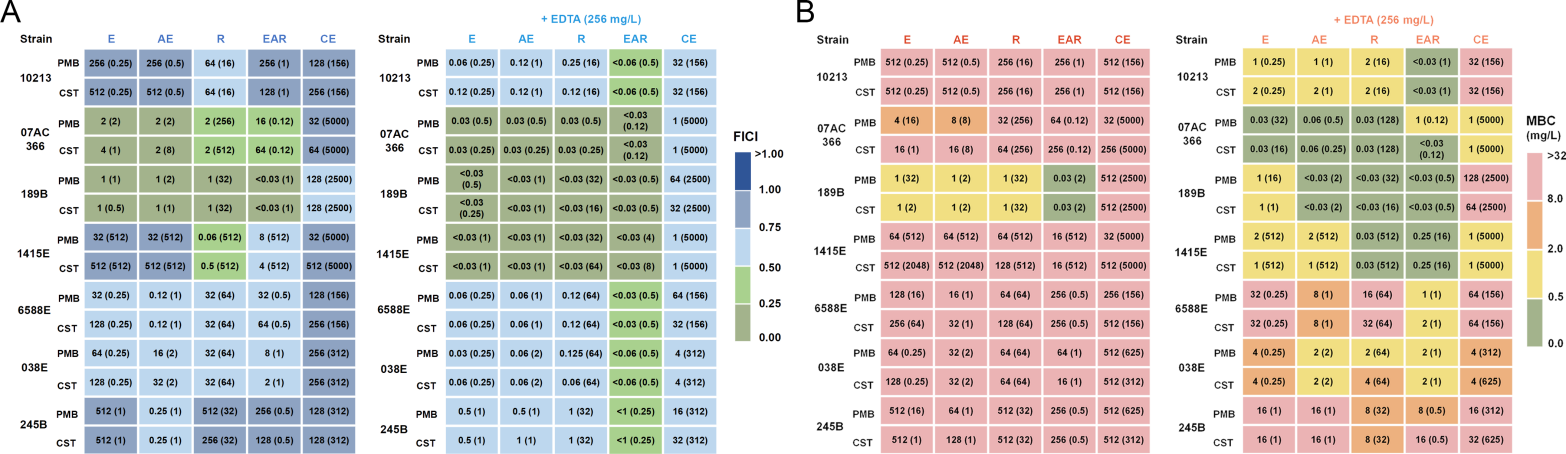
Antibacterial and bactericidal activity of combination treatment against polymyxin-resistant *A. baumannii* strains. **(A)** MICs (mg/L) against *A. baumannii* strains by PMB and CST combined with E, AE, R, EAR, or CE, without EDTA (left panel) and with 256 mg/L EDTA supplementation (right panel). The values in parentheses represent optimal concentrations of E, AE, R, EAR, and CE (mg/L). The color legend represents the calculated FICI based on the MIC values provided in **Table 1**. FICI < 0.5 indicates synergism, 0.5 ≤ FICI < 1 indicates additivity. **(B)** MBCs (mg/L) against *A. baumannii* strains by PMB and CST combined with E, AE, R, EAR, or CE, without EDTA (left panel) and with 256 mg/L EDTA supplementation (right panel). The values in parentheses represent optimal concentrations of E, AE, R, EAR, and CE (mg/L). The color legend corresponds to varying levels of drug sensitivities. E, emodin; AE, aloe emodin; R, rhein; EAR, emodin/aloe emodin/rhein in a mass ratio of 1:1:1; CE, crude extract of *A. barbadensis* by Lac-Glu solvent; PMB, polymyxin B; CST, colistin; FICI, fractional inhibitory concentration index.

Acknowledging the well-established potential of natural products to synergize with polymyxins and enhance their antibacterial effects against multidrug-resistant bacteria (Lin et al., 2021), we attempted to assess the effects of aloe emodin, emodin, rhein, EAR and crude extracts (LacGlu-AB as the representative) when combined with polymyxins. Through checkerboard assay, significant synergistic effects (FICI < 0.5) of the tested compounds were observed that resulted for enhancing the sensitivity of certain polymyxin-resistant *A. baumannii* strains to PMB and CST (**Figure 2A**-left panel and **Supplementary Table 1**). Specifically, exposure to a 1 mg/L concentration of EAR reduced the MIC of polymyxins against *A. baumannii* 189B from an initial value exceeding 512 mg/L to below 0.03 mg/L (**Table 1**; **Supplementary Table S1**). However, it was noteworthy that aside from strain 189B, the MBC of polymyxins against other *A. baumannii* strains remained considerably high, ranging from 4 to 512 mg/L (**Figure 2B**-left panel). This outcome suggested the necessity for further optimization strategies to achieve higher antibacterial activities across diverse strains of polymyxin-resistant *A. baumannii*. To facilitate this objective, EDTA was chosen for incorporation due to its previously indicated capability to disrupt the Gram-negative bacterial outer membrane by chelating Mg^2+^ and Ca^2+^, consequently increasing the permeability of the outer membrane (Nabil et al., 2000).

Using *A. baumannii* strain 1415E as an example, a notable decrease in the effective concentration of polymyxins was observed when combined with EDTA and aloe emodin, emodin, rhein, EAR, or crude extracts, respectively (**Supplementary Table 2**). Particularly, in the presence of 256 mg/L EDTA, the MIC of colistin combined with EAR against the strain 1415E decreased by up to 16,384-folds. Therefore, the antibacterial and bactericidal activities of polymyxins in combination with aloe emodin, emodin, rhein, EAR, or crude extracts against seven polymyxin-resistant *A. baumannii* strains were further analyzed in the presence of 256 mg/L EDTA. As expected, the results revealed that the MIC of combination treatments achieved a decrease of 4 to 16384 folds compared to polymyxins treatments (**Figure 2A**; **Supplementary Table 3**), and the MBC of combination treatments reduced to 0.03 ~ 64 mg/L (**Figure 2B**; **Supplementary Table 3**). Remarkably, EAR exhibited unique advantages when combined with 256 mg/L EDTA and polymyxins, leading to a significant reduction in both MIC and MBC, and demonstrating clear synergistic effects (FICI < 0.5). Therefore, subsequent research primarily focuses on combination with the EAR mixture rather than aloe emodin, emodin, rhein or crude extracts.

**Figure 3:**
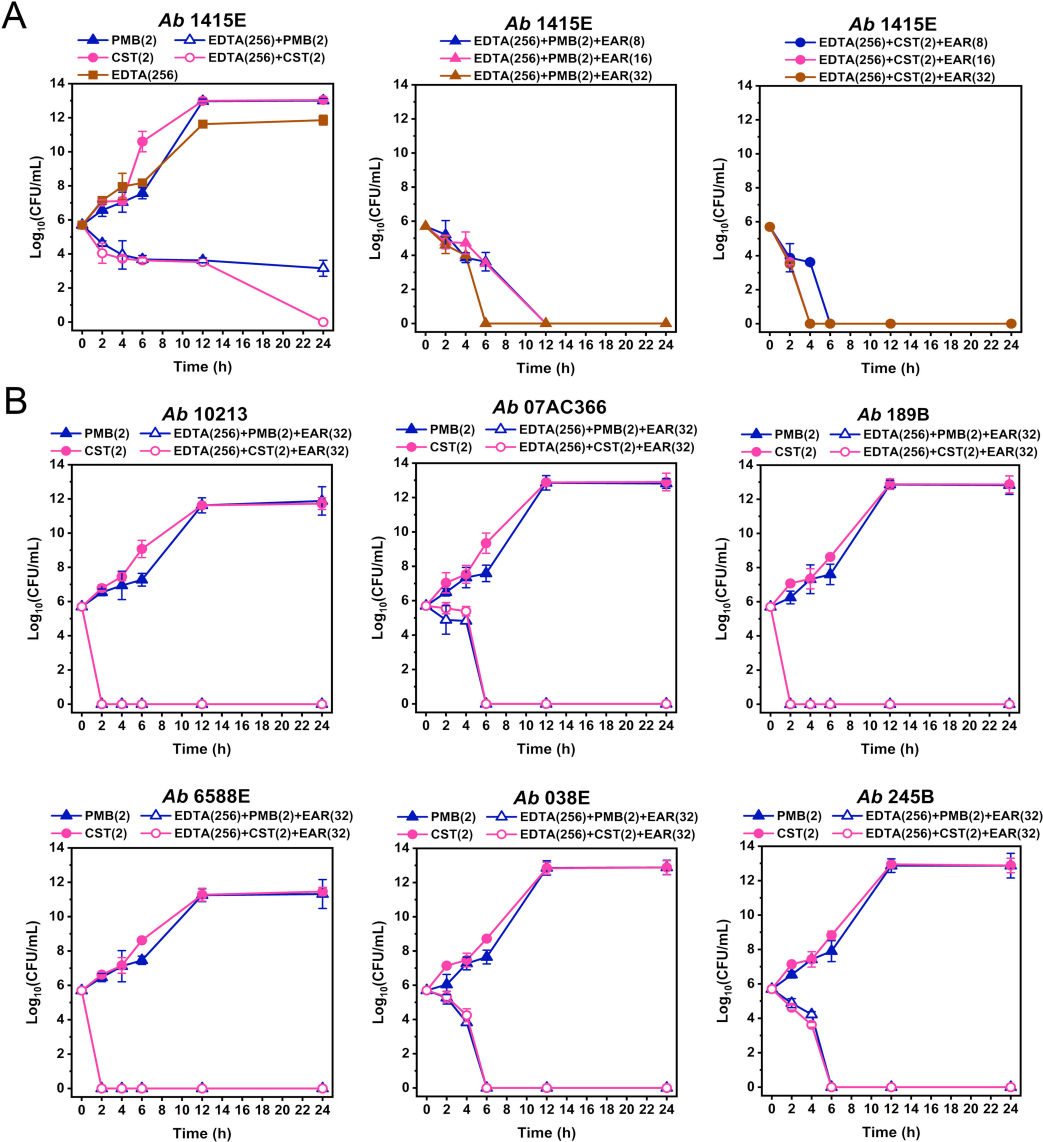
Time-kill curves of different drug combinations against polymyxin-resistant *A. baumannii* strains. **(A)** Time-kill curves of PMB (2 mg/L, middle panel) and CST (2 mg/L, right panel) combined with EDTA (256 mg/L) and EAR (ranging from 8 to 32 mg/L) against *A. baumannii* 1415E. PMB, CST, and EDTA are used alone or in combinations as controls (left panel). **(B)** Time-kill curves of PMB (2 mg/L) and CST (2 mg/L) combined with EDTA (256 mg/L) and EAR (32 mg/L) against six polymyxin-resistant strains of *A. baumannii*. PMB and CST are used as controls. Data are presented as mean ± standard deviation (n = 3). EAR, emodin/aloe emodin/rhein in a mass ratio of 1:1:1; PMB, polymyxin B; CST, colistin.

Through comprehensive analysis of MBC data (**Figure 2**; **Supplementary Tables 1-3**), the concentrations of EDTA and PMB (or CST) in the combination treatment were determined to be 256 mg/L and 2 mg/L, respectively. To determine the optimal working concentration of EAR in the combination treatment, gradient concentrations of EAR at 8 mg/L, 16 mg/L, and 32 mg/L was supplemented in the combination to measure its time-kill curve against *A. baumannii* 1415E, respectively. As shown in **Figure 3A**, 256 mg/L EDTA and 2 mg/L polymyxins had no obvious impact on the growth of 1415E when used individually. Combining EDTA with polymyxins significantly inhibited the growth of 1415E, and the total killing of 1415E was observed in the EDTA-CST group after 24 h of treatment. When EAR was added in the combination, the bactericidal efficiency against 1415E was greatly enhanced, especially the supplement of 32 mg/L EAR achieved the complete sterilization within 6 h and 4 h in the PMB and CST group, respectively. Therefore, 256 mg/L EDTA, 2 mg/L PMB or CST, and 32 mg/L EAR were used in the subsequent combination treatment. As expected, the combination treatments showed rapid killing effects against all the tested polymyxin-resistant strains in a short period of time less than 6 h (**Figure 3B**). Notably, *A. baumannii* 10213, 189B, and 6588E were completely eradicated within 2 h, meeting the objective of rapid sterilization.

### 3.3 Combination treatment suppressed the emergence of polymyxin resistance to polymyxin-sensitive *A. baumannii* strains

**Figure 4:**
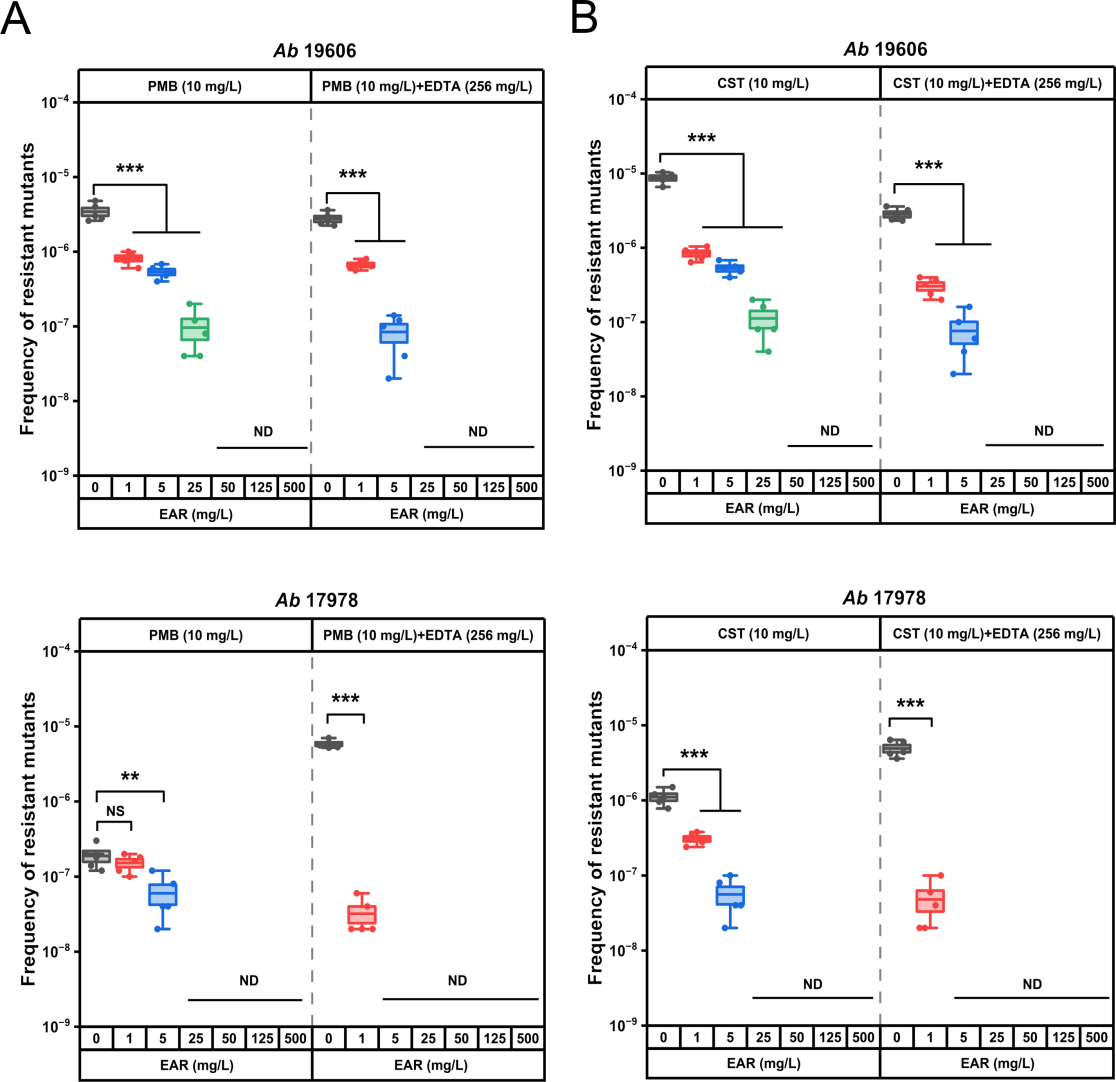
*In vitro* assessment of resistant mutation frequency of polymyxin B and colistin alone or in combination with EDTA and EAR against polymyxins-sensitive strains of *A. baumannii* ATCC 19606 and 17978. Box plots represent the resistant mutation frequency of ATCC 19606 (first panel) and 17978 (second panel) to PMB **(A)** and CST **(B)** after exposure to 10 mg/L polymyxins alone or supplemented with EDTA (256 mg/L) and EAR (ranging from 0 to 500 mg/L). Each dot represents the value of an individual measurement (n = 5), and the mean value is shown by the line. EAR, emodin/aloe emodin/rhein mass ratios of 1:1:1; PMB, polymyxin B; CST, colistin. **, *P* < 0.01, ***, *P* < 0.001; ND, not detected; NS, not significant.

High-concentration polymyxin treatment (≥4 MIC) *A. baumannii* populations evolved high-level and fixed resistance (Zhao et al., 2022). Given that EDTA in combination with EAR restored polymyxins sensitivity in polymyxin-resistant *A. baumannii*, was it possible for this combination therapy to further increase polymyxins sensitivity in polymyxins-sensitive *A. baumannii*? To answer this question, determination of the frequency of polymyxin-resistant mutation were performed with both EAR and EDTA in combination with the polymyxins and reference bacterial strains *A. baumannii* ATCC 19606 and 17978. The frequency of resistant mutations in strains 19606 and 17978 to polymyxins was assessed under the selective pressure of 10 mg/L PMB or CST (**Figure 4**). The polymyxins-resistant mutation frequency was determined to be 10^-6^ ~ 10^-5^ for strain 19606 and 10^-7^ ~ 10^-6^ for strain 17978. Notably, the presence of EAR led to a significant reduction in the mutation frequency of strains 19606 and 17978 to polymyxins in a concentration-dependent manner (*P* < 0.001). Specifically, when the concentration of EAR exceeded or equaled 50 mg/L, the polymyxins-resistant mutation frequency in strain 19606 dropped below 10^-9^. Moreover, in the presence of 256 mg/L EDTA, EAR at a concentration of 25 mg/L was sufficient to achieve a polymyxins-resistant mutation frequency below 10^-9^. Analogously, a concentration of EAR equal to or exceeding 25 mg/L was observed to attenuate the mutation frequency conferring polymyxins resistance in strain 17978 to below 10^-9^. Furthermore, in the presence of 256 mg/L EDTA, the required EAR concentration was reduced to 5 mg/L.

### 3.4 The drug combination exhibited low cytotoxicity and accelerated the healing of skin wounds in mice infected with polymyxin-resistant *A. baumannii* 1415E

**Figure 5:**
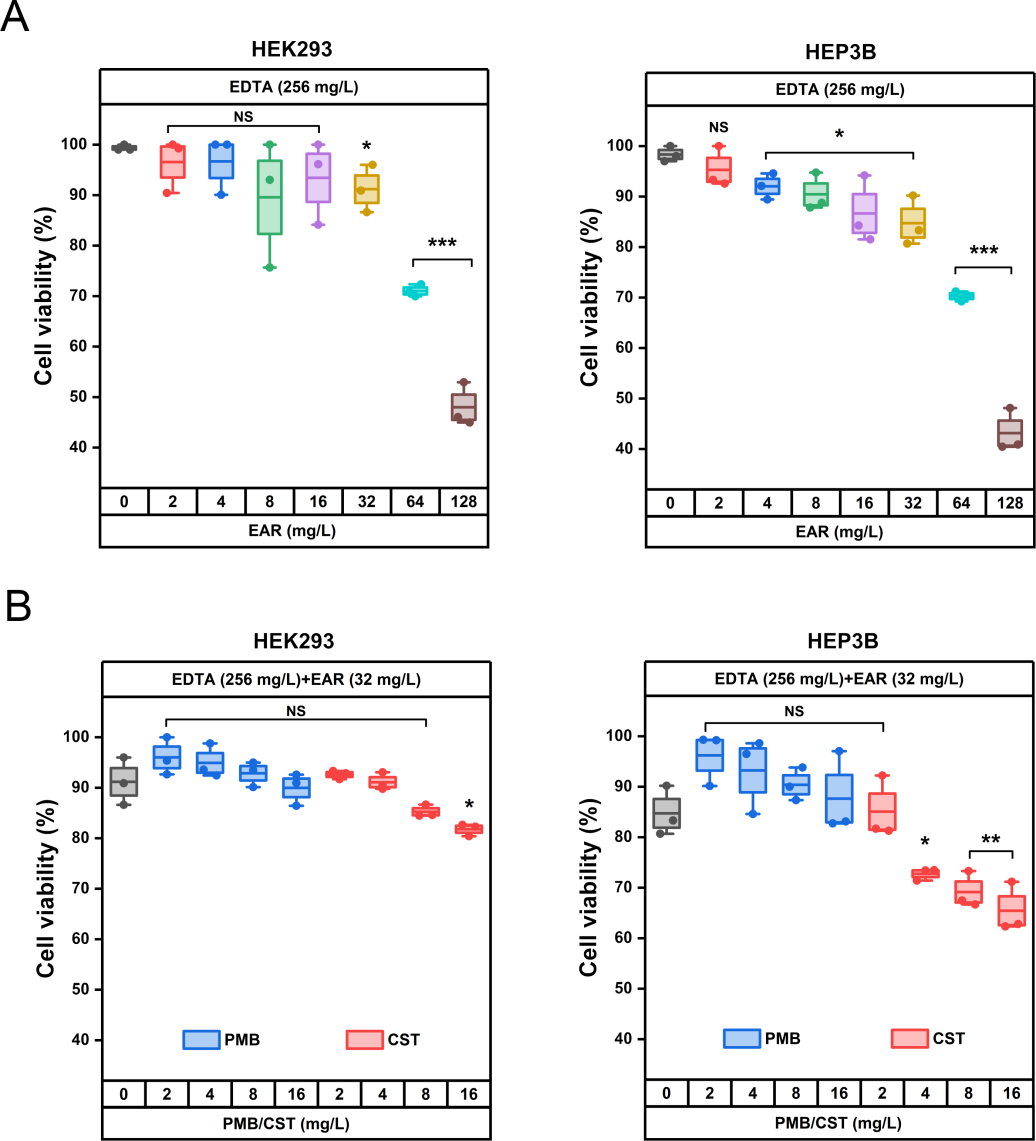
Cytotoxicity evaluation of the drug combination using HEK293 and HEP3B cells *in vitro*. **(A)** Effects of 256 mg/L EDTA supplemented with different concentrations of EAR (ranging from 0 to 128 mg/L) on the viability of HEK293 cells (left) and HEP3B cells (right). **(B)** Effects of different concentrations of PMB (blue boxes, ranging from 0 to 16 mg/L) and CST (red boxes, ranging from 0 to 16 mg/L) combining 256 mg/L EDTA and 32 mg/L EAR on the viability of HEK293 cells (left) and HEP3B cells (right). Each dot represents the value of an individual measurement (n = 3), and the mean value is shown by the line. EAR, emodin/aloe emodin/rhein in a mass ratio of 1:1:1; PMB, polymyxin B; CST, colistin; *, *P* < 0.05, **, *P* < 0.01, ***, *P* < 0.001; NS, not significant.

**Figure 6:**
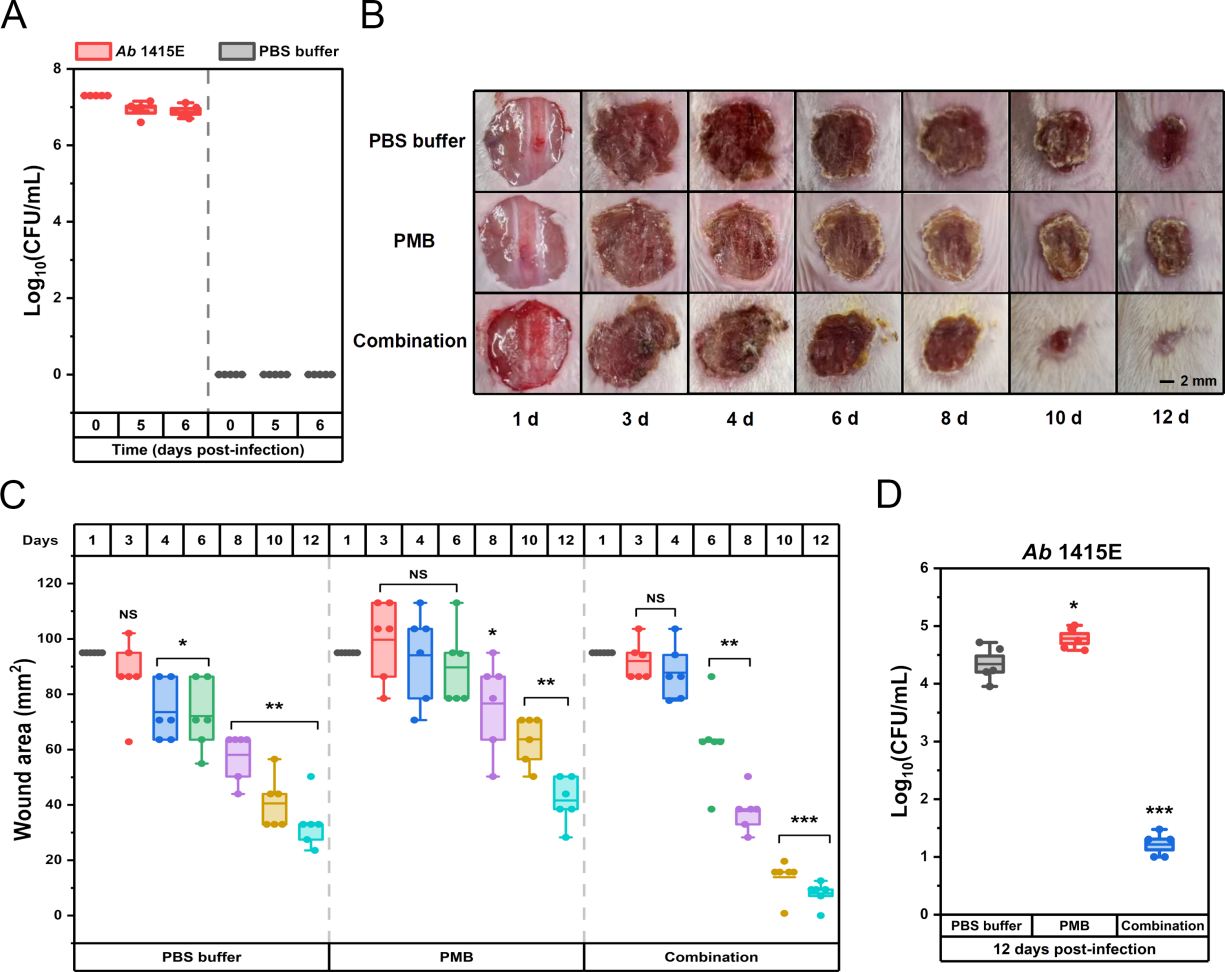
Therapeutic potential of combination therapy for polymyxin-resistant *A. baumannii* 1415E induced skin infections in BALB/C mice. **(A)** The bacterial burden of *A. baumannii* 1415E in infected wounds by counting CFUs at the 0^th^, 5^th^, and 6^th^ d post-infection (n = 5). **(B)** Representative photographs of the infected wounds on mice skin captured at the 1^st^, 3^rd^, 4^th^, 6^th^, 8^th^, 10^th^, and 12^th^ d post-treatments with PBS buffer, PMB (2 mg/mL) or drug combination (32 mg/kg EDTA+ 4 mg/kg EAR+ 2 mg/kg PMB). Scale bar represents 2 mm. **(C)** Box plots represent the measured wound areas (mm^2^) at the 1^st^, 3^rd^, 4^th^, 6^th^, 8^th^, 10^th^, and 12^th^ d post-treatments (n = 6). **(D)** The bacterial burden of *A. baumannii* 1415E in infected wounds by CFU count at 12 days post-treatments (n = 6). The group treated with PBS buffer serves as the mock control. Each dot represents the value of an individual measurement, and the mean value is shown by the line. *, *P* < 0.05, **, *P* < 0.01, ***, *P* < 0.001. EAR, emodin/aloe emodin/rhein in a mass ratio of 1:1:1; PMB, polymyxin B; NS, not significant.

Cytotoxicity assays, pivotal in assessing the clinical development potential of therapeutic strategies, were performed on kidney (HEK293) and liver (HEP3B) cell line to evaluate the safety profile of the drug combination. As shown in **Figure 5A**, in the presence of 256 mg/L EDTA, EAR not exceeding 32 mg/L were found to have no significant effect on the viability of HEK293 cells, while doses ranging from 4 to 32 mg/L of EAR were observed to result in a marginal reduction (ranging from 6.31% to 13.60%, *P* < 0.05) in the viability of HEP3B cells. Upon combining 256 mg/L EDTA and 32 mg/L EAR with polymyxins (**Figure 5B**), various concentrations of PMB (ranging from 2 to 16 mg/L) showed no significant influence on the viability of either HEK293 or HEP3B cells. In contrast, higher concentrations of CST (16 mg/L) led to a notable decrease in the viability of HEK293 cells by 9.39% and of HEP3B cells, ranging from 11.96% (at 4 mg/L CST) to 19.29% (at 16 mg/L CST), which is consistent with the previous report that CST was cytotoxic at relatively high concentrations (Ritesh and Arun, 2018). Given the comparatively lower cell toxicity observed with PMB compared to CST, the combination strategy involving PMB was chosen for subsequent mouse skin infection treatment. Considering the excellent performance of *Aloe* in treating skin diseases and promoting skin wound healing (Liang et al., 2021), the skin wound infection in mice was used to evaluation efficacy of the drug combination. Due to its representative nature characterized by strong resistance to polymyxins and EAR *in vitro* (**Table 1**), the clinically isolated *A. baumannii* 1415E was employed to establish the infection. On the 5^th^ and 6^th^ d post-infection (**Figure 6A**), the bacterial burden of 1415E at the wound site of the mice stabilized at approximately 8×10^6^ CFUs/mL, indicating the successful establishment of the mouse skin wound infection, despite that the bacterial load slightly decreased compared to the initial load (2 × 10^7^ CFUs/mL). After 12 d of continuous combination therapy (32 mg/kg EDTA + 4 mg/kg EAR + 2 mg/kg PMB), the wound area of the mice gradually decreased from the initial 94.98 mm^2^ to 7.98 ± 3.91 mm^2^, showing a trend of gradual healing (**Figures 6B, C**). At the same time, the wound healing both in the mock treatment (PBS buffer) group and the PMB monotherapy group (2 mg PMB/kg) was remarkably slower, with wound areas of 33.36 ± 8.33 mm^2^ and 41.61 ± 7.66 mm^2^, respectively. Moreover, the colony counting of the wound skin tissue of the mice was performed after 12 d of treatment, and the results (**Figure 6D**) showed that the bacterial burden of the combination therapy group was 10 ~ 30 CFUs/mL, significantly (*P* < 0.001) lower than that of the PMB monotherapy group (4.20 × 10^4^ ~ 1.03 × 10^5^ CFUs/mL) and the mock treatment group (9.00 × 10^3^ ~ 5.20 × 10^4^ CFUs/mL). The results indicated that the drug combination exhibited great potential to treat the skin infections caused by polymyxin-resistant *A. baumannii*.

## 4 Discussion

Polymyxins, serving as the “last-line” therapeutics for combating multidrug-resistant *A. baumannii* infections, face a formidable challenge attributed to the emergence and worldwide dissemination of polymyxin-resistant *A. baumannii* strains, thereby posing a major public health threat (Shi et al., 2024). The occurrence of treatment failures with polymyxin monotherapies has prompted the adoption of polymyxin combination therapy in clinical practice, which is increasingly proposed as a potential strategy to enhance antimicrobial activity and mitigate the development of resistance (Lenhard et al., 2016). As crucial reservoirs of novel antimicrobial compounds boasting diverse mechanisms of action, natural products derived from medicinal plants have emerged as potent candidates for combating antimicrobial-resistant pathogens, either independently or when combined with conventional antibiotics (Zhang et al., 2020; Song et al., 2021; Arip et al., 2022). Aloe stands out prominently for its renowned properties and various therapeutic activities, including anti-bacterial, anti-viral, anti-cancer effects, as well as immunoregulative and hepatoprotective properties (Gao et al., 2018). Aloe exerts antibacterial activity through a multi-target mode of action, with notable efficacy against Gram-positive bacteria such as *Staphylococcus aureus* and *Staphylococcus epidermidis*, suggesting its potential application in the pharmaceutical field (Ushasree et al., 2024).

NADES, as an emerging green solvent, were increasingly applied in the pharmaceutical, cosmetics, and food industries. These solvents provided several advantages for natural product extraction, including ease of preparation, high solubility, enhanced conductivity, biodegradability, and low toxicity. These properties not only facilitated more in-depth exploration of natural compounds but also aligned with environmental and sustainable development objectives (Hikmawanti et al., 2021; Chen et al., 2023; Villa et al., 2024). In this study, the extracts of AA and AB by NADES demonstrated superior inhibitory activity against *A. baumannii* COLR 10213 in comparison to those of AF and AL. This enhanced activity was probably due to the higher anthraquinone content in the extracts of AA and AB, a phenomenon supported by the established ability of NADES to enhance the solubility of anthraquinones, thereby improving the extraction efficiency of these compounds (Wu et al., 2018). The higher anthraquinone content in AA and AB compared to AF and AL might be attributed to the intrinsic properties of the respective *Aloe* species. Additionally, the variation in anthraquinone content might also be associated with other factors, such as the regulation of secondary metabolites in response to environmental stress (Breaud et al., 2022; Maliehe et al., 2023). The superior activity of EAR compared to its monomers possibly resulted from the functional group variations among the monomers, corresponding to different targets and thereby facilitating synergistic interactions and enhancing overall efficacy (Hu et al., 2023).

The strain-specific antibacterial and bactericidal activities exhibited by anthraquinone compounds from *Aloe* among these polymyxins-resistant *A. baumannii* strains might be attributed to the diverse mechanisms of polymyxin resistance, leading to alterations in the outer membrane and variations in outer membrane permeability (Nang et al., 2021). The variability in polymyxins resistance mechanisms among these polymyxins-resistant *A. baumannii* strains might be inferred from the characteristic inter-strain differences in their susceptibility to polymyxins (**Table 1**). The outer membrane of Gram-negative bacteria was an asymmetric bilayer consisting an inner leaflet of phospholipids and an outer leaflet that served as a crucial barrier against harmful components like antimicrobial agents (Nikaido, 2003). The permeability barrier of the outer membrane impeded the entry of hydrophobic substances from *Aloe*, thus research on the antibacterial activity of *Aloe* mainly focused on Gram-positive bacteria (Ushasree et al., 2024). EDTA was frequently reported to increase bacterial membrane permeability and combine with polymyxins for anti-infective therapy (Hamel et al., 2021). Therefore, we attempted to incorporate EDTA into the process of combating *A. baumannii*.

EDTA exhibited high MIC and MBC values against *A. baumannii*, indicating a lack of antibacterial activity, while EAR exhibited a low MIC but high MBC values (**Table 1**), suggesting weak bactericidal activity (**Figure 2**; **Supplementary Tables 1–3**). The combination of EAR and EDTA still exhibited a lack of bactericidal activity (**Supplementary Table 3**), suggesting that anthraquinone compounds possibly acted as inhibitors affecting the metabolic processes of *A. baumannii*. The combination of EAR and EDTA restored the sensitivity of polymyxins-resistant *A. baumannii* to polymyxins and synergistically enhanced the bactericidal efficacy of polymyxins (**Figures 2**, **3**). Given the weak bactericidal activity of EAR and EDTA, the bactericidal activity exhibited by the combination strategy against polymyxins-resistant *A. baumannii* was probably contributed by polymyxins. The emergence of this phenomenon was plausibly attributed to the interference induced by EAR and EDTA, which disrupted the intrinsic resistance mechanisms associated with polymyxins in these strains, thereby reinstating their susceptibility to polymyxins. Rhein demonstrated antimicrobial activity against *Staphylococcus xylosus* by interfering with bacterial energy metabolism, inducing reactive oxygen species (ROS) production, and causing cell membrane and DNA damage (Li et al., 2024). This suggested that EAR might operate via a similar mechanism against *A. baumannii*. Higher ROS levels were linked to increased cell membrane permeability (Seyedjavadi et al., 2020). As a metal ion chelator, EDTA could bind to divalent cations such as Mg^2+^ and Ca^2+^, which were critical for maintaining the structural stability of the bacterial outer membrane (Nabil et al., 2000). This chelation further enhanced membrane permeability, leading to the induction of even higher levels of ROS. ROS could act as a double-edged sword, such as being essential for regulating cellular signaling pathways and physiological processes at low levels, while disrupting the redox balance, pushing bacteria into a state of oxidative stress that eventually leads to cell death at high levels. To mitigate ROS-induced oxidative damage, bacteria have evolved an inducible antioxidant defense system (Lu and Holmgren, 2014; Zhao and Drlica, 2014). Research has shown that polymyxin not only disrupting the membrane but also significantly increases ROS levels in *A. baumannii* (Fu et al., 2024). However, the use of polymyxin alone might not induce overproduction of ROS, or the ROS induced by polymyxin might be neutralized by the bacterial antioxidant defense system in polymyxin-resistant *A. baumannii*. EAR and EDTA, which might increase membrane permeability, could assist polymyxin in generating higher levels of ROS. This combined effect could overwhelm the bacterial antioxidant defense system, leading to irreversible ROS damage, accelerating bacterial death, and achieving rapid bactericidal effects through combination therapy. However, the precise underlying mechanism remained elusive.

In addition to polymyxins-resistant strains, the combination of EAR and EDTA also enhanced the susceptibility of polymyxins to polymyxins-sensitive strains, such as ATCC 19606 and 17978. The mutational frequency detection revealed that strains ATCC 19606 and 17978 exhibited a propensity for polymyxins-resistant mutations (**Figure 4**), which was consistent with prior literature findings (He et al., 2024). However, co-administration of EAR and EDTA markedly reduced the frequency of polymyxins-resistant mutations, providing further evidence of their ability to sensitize *A. baumannii* to polymyxins.

EDTA was primarily used in clinical settings for the treatment of heavy metal ion poisoning, such as lead poisoning (Warsi et al., 2024). Intravenous administration of EDTA could induce renal toxicity, particularly in patients with renal diseases or those receiving high doses of the drug. Additionally, EDTA could chelate other ions, potentially leading to deficiencies resulting in health issues like anemia, cardiac arrhythmias, and neurological disorders. Therefore, Ca-EDTA was typically recommended for intravenous injection due to its significantly reduced toxicity, despite comparatively lower efficacy (Wannigama et al., 2023). Anthraquinone derivatives, which were present in various medicinal plants, had been increasingly associated with potential safety issues as their applications expanded, particularly hepatotoxicity and nephrotoxicity due to their high doses or improper use. Although the necessity for risk assessment of anthraquinone derivatives was emphasized, these compounds, as lead compounds, would still have attractive application prospects if structural modification was performed in subsequent research (Dong et al., 2019; Liu et al., 2020; Semwal et al., 2021). The reported heightened risk of nephrotoxicity emerged as the primary limiting factor in the clinical utilization of polymyxins (Wagenlehner et al., 2021). In our study, CST exhibited relatively greater cellular toxicity in the human hepatic and renal cell lines than PMB (**Figure 5**), which was consistent with previous reports (Ismail et al., 2018; Paul et al., 2018; Mattos et al., 2019).

To determine the appropriate therapeutic concentration for the mouse skin infection model, the clinically recommended human dose of EDTA, which was 25 mg/kg (Wei et al., 2023), was used as a reference. Applying the animal equivalent dose calculation formula based on body surface area (Nair and Jacob, 2016), the equivalent dose for mice was estimated to be approximately 307.5 mg/kg. Similarly, the International Consensus Guidelines for the Optimal Use of Polymyxins recommend a clinical dose of 2.0 to 2.5 mg/kg for PMB in humans (Tsuji et al., 2019), leading to an equivalent mouse dose of about 24.6 to 30.8 mg/kg. Due to concerns about drug toxicity and the tolerance of the mice, these theoretical doses were cautiously reduced by approximately tenfold. Taking into account the MBC and *in vitro* cytotoxicity, the therapeutic dose for EDTA and PMB were ultimately set at 32 mg/kg and 2 mg/kg, respectively. For the therapeutic dose of EAR, where standardized guidelines were lacking, recent *in vivo* studies on compounds like Emodin (>4 mg/kg, Chen et al., 2022), Aloe-emodin (>4 mg/kg, Yan et al., 2023), and Rhein (>4 mg/kg, Liu et al., 2023) were consulted. Based on the MBC and *in vitro* cytotoxicity, a safe dose of 4 mg/kg for EAR in mice studies was established. In summary, this approach ensured that the treatment remained within safe limits while minimizing antibiotic use to reduce the potential for resistance development. Ultimately, the combination of the less toxic PMB with EDTA and EAR was selected for treating mouse skin infections. Given that the infecting *A. baumannii* 1415E strain was resistant to polymyxins, the efficacy of PMB monotherapy did not significantly differ from that of the PBS buffer control, as indicated by similar wound healing trends in both groups (**Figures 6B, C**). Wounds in the PMB monotherapy and PBS buffer groups healed slowly with a reduction in bacterial load, possibly due to the response of the mice’s own immune system. With the addition of anthraquinone compounds (EAR), wound healing significantly accelerated (**Figure 6D**), which was consistent with reports that *Aloe* played an important role in promoting wound healing (Liang et al., 2021).

## 5 Conclusion

Anthraquinone compounds represented by aloe emodin, emodin, and rhein, when used in combination with EDTA, could sensitize *A. baumannii* to polymyxins, thereby restoring the polymyxins-sensitive phenotype in polymyxins-resistant *A. baumannii*. In addition to enhancing antibacterial activity, this combination strategy significantly increased the bactericidal efficiency of polymyxins. Under this combined strategy, the resistant mutation frequency of polymyxins-sensitive *A. baumannii* to polymyxins was suppressed. This combination strategy exhibited less toxicity to human hepatic and renal cell lines and accelerated the healing of mouse skin wounds infected by polymyxins-resistant *A. baumannii*. In summary, our findings provided a potential therapeutic strategy for infections caused by polymyxins-resistant *A. baumannii*.

## Data Availability

The original contributions presented in the study are included in the article/**Supplementary Material**. Further inquiries can be directed to the corresponding authors.
