# Supplementary material for: Combination of aloe emodin, emodin, and rhein from Aloe with EDTA sensitizes the resistant Acinetobacter baumannii to polymyxins
Source: Front Cell Infect Microbiol. 2024 Sep 13;14:1467607. doi: 10.3389/fcimb.2024.1467607 (PMC11428196; doi:10.3389/fcimb.2024.1467607)
Supplement: Supplementary file 1 [file DataSheet1.docx]

Supplementary Material


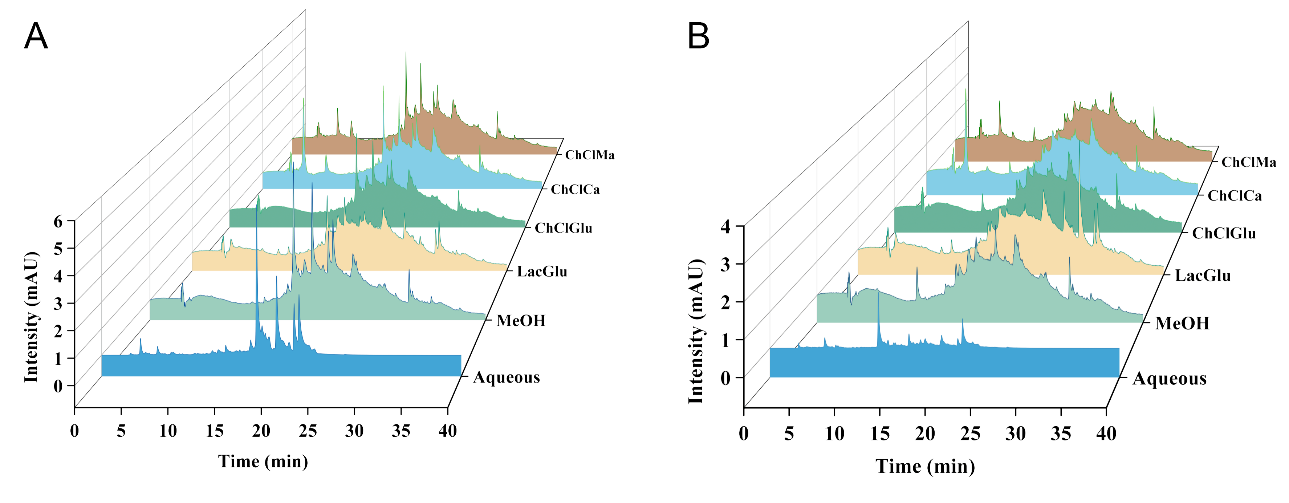


**Supplementary Figure 1.** Comparison of high-performance liquid chromatography (HPLC) fingerprint chromatograms of crude extracts from *Aloe arborescens* (A) and *Aloe barbadensis* (B) using various solvent systems at 254 nm wavelength. MeOH, methanol; ChClGlu, choline chloride/ D-glucose in a molar ratio of 1:1; ChClMa, choline chloride/ malic acid in a molar ratio of 2:1; ChClCa, choline chloride/ citric acid in a molar ratio of 2:1; LacGlu, lactic acid/ D-glucose in a molar ratio of 5:1.


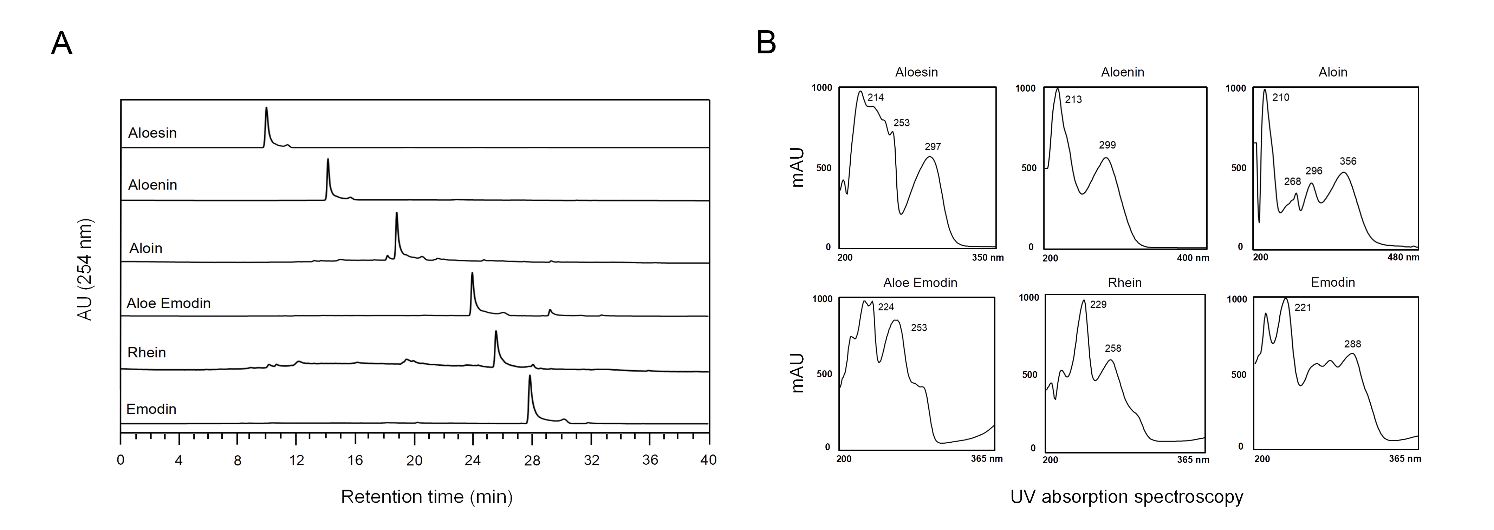


**Supplementary Figure 2.** HPLC analysis of aloesin, aloenin, aloin, aloe emodin, rhein, and emodin. (A) Comparison of retention times of 6 standard substances monitored at 254 nm wavelength. (B) Ultraviolet absorption spectroscopy of 6 standard substances.


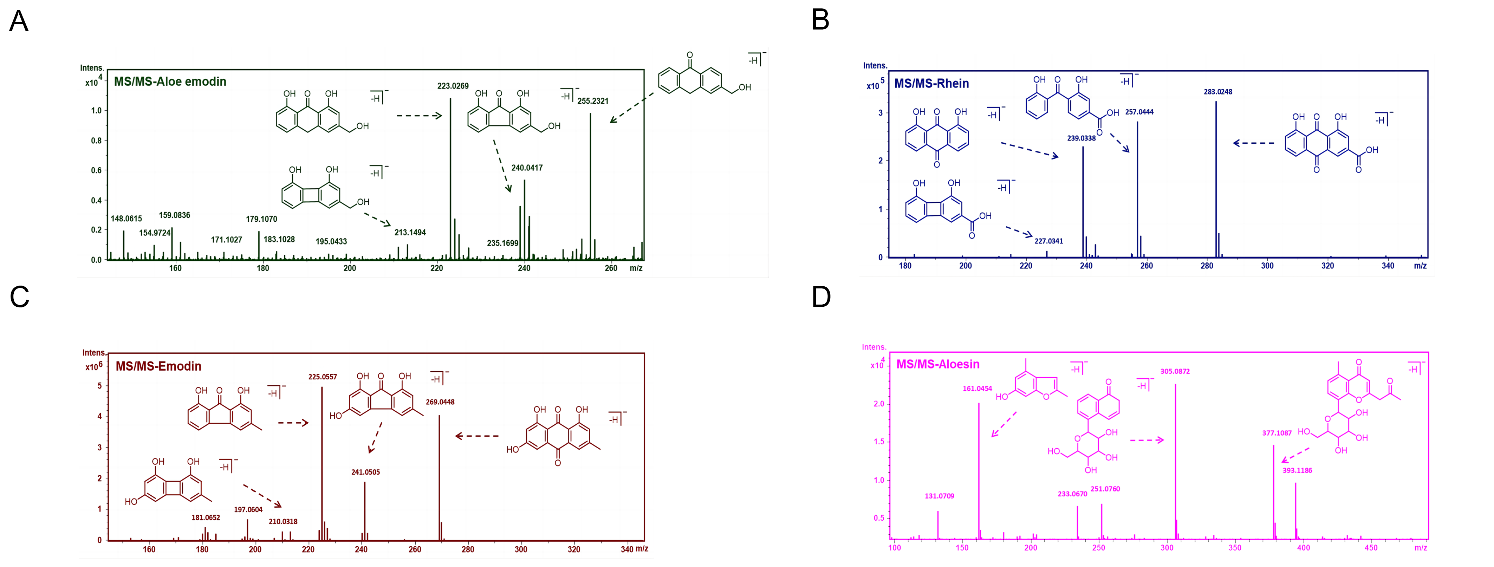


**Supplementary Figure 3.** High-resolution secondary mass spectrometry analysis of peak 1 (A), peak 2 (B), peak 3 (C), and peak 4 (D) in FIG 1C and D, which are identified as aloe emodin, rhein, emodin, and aloesin, respectively.


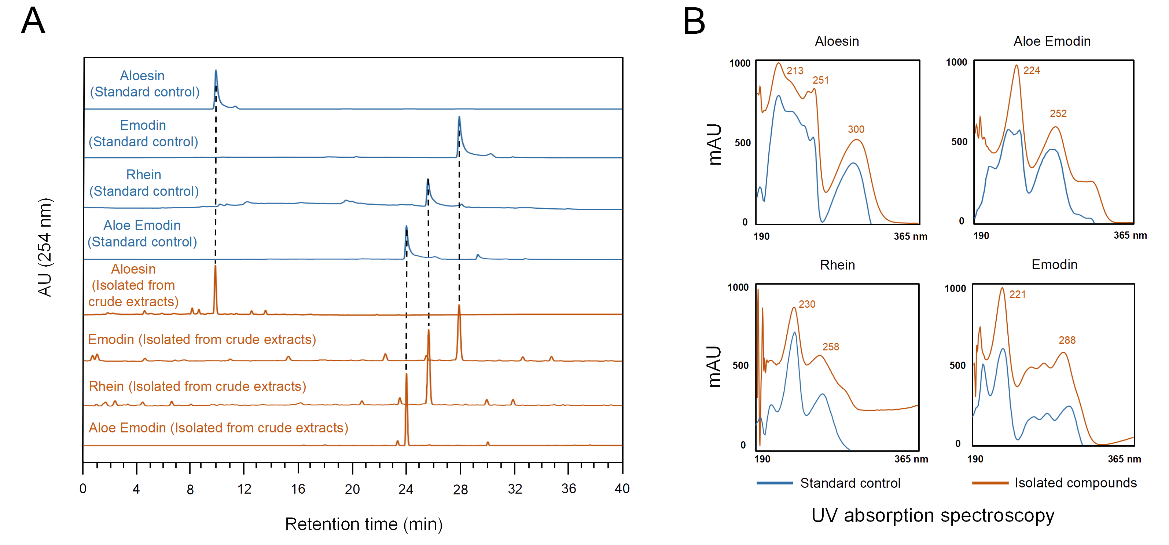


**Supplementary Figure 4.** Comparative analysis of isolated peaks 1-4 in FIG 1C&D with the standard compounds by HPLC. (A) Chromatogram alignment of isolated with standard compounds monitored at 254 nm wavelength. (B) Ultraviolet absorption spectroscopy alignment of isolated with standard compounds.

**Supplementary Table 1.** The antibacterial and bactericidal activity of PMB and CST against polymyxin-resistant *A. baumannii* strains in the present of E, AE, R, EAR, or CE.

| Compounds | Contents | 10213 | 07AC366 | 189B | 1415E | 6588E | 038E | 245B |
| --- | --- | --- | --- | --- | --- | --- | --- | --- |
| Polymyxin B  +E^1^ | MIC (mg/L) | 256 + 0.25 | 2 + 2 | 1 + 1 | 32 + 512 | 32 + 0.25 | 64 + 0.25 | 512 + 1 |
|  | FICI^2^ | >0.75, <1 | >0, <0.03 | >0, <0.003 | >0.75, <1 | >0.5, <0.75 | >0.5, <0.75 | >0.75, <1 |
|  | Fold change in MIC decrease^2^ | >2 | 32 | >512 | 2 | 8 | >8 | >1 |
|  | MBC (mg/L) | 512 + 0.25 | 4 + 16 | 1 + 32 | 64 + 512 | 128 + 16 | 64 + 0.25 | 512 + 16 |
| Colistin  +E^1^ | MIC (mg/L) | 512 + 0.25 | 4 + 1 | 1 + 0.5 | 512 + 512 | 128 + 0.25 | 128 + 0.25 | 512 + 1 |
|  | FICI^2^ | >0.75, <1 | >0, <0.02 | >0, <0.002 | >0.75, <1 | >0.5, <0.75 | >0.5, <0.75 | >0.75, <1 |
|  | Fold change in MIC decrease^2^ | >1 | 64 | >512 | >1 | 4 | 4 | 1 |
|  | MBC (mg/L) | 512 + 0.25 | 16 + 1 | 1 + 2 | 512 + 2048 | 256 + 64 | 128 + 0.25 | 512 + 1 |
| Polymyxin B  +AE^1^ | MIC (mg/L) | 256 + 0.5 | 2 + 2 | 1 + 2 | 32 + 512 | 0.125 + 1 | 16 + 2 | 0.25 + 1 |
|  | FICI^2^ | >0.75, <1 | >0, <0.03 | >0, <0.01 | >0.75, <1 | >0.5, <0.75 | >0.5, <0.75 | >0.5, <0.75 |
|  | Fold change in MIC decrease^2^ | >2 | 32 | >512 | 2 | 2048 | >32 | >2048 |
|  | MBC (mg/L) | 512 + 0.5 | 8 + 8 | 1 + 2 | 64 + 512 | 16 + 1 | 32 + 2 | 64 + 1 |
| Colistin  +AE^1^ | MIC (mg/L) | 512 + 0.5 | 2 + 8 | 1 + 1 | 512 + 512 | 0.125 + 1 | 32 + 2 | 0.25 + 1 |
|  | FICI^2^ | >0.75, <1 | >0, <0.02 | >0, <0.06 | >0.75, <1 | >0.5, <0.75 | >0.5, <0.75 | >0.5, <0.75 |
|  | Fold change in MIC decrease^2^ | >1 | 128 | >512 | >1 | 4096 | 16 | 2048 |
|  | MBC (mg/L) | 512 + 0.5 | 16 + 8 | 1 + 2 | 512 + 2048 | 32 + 1 | 32 + 2 | 128 + 1 |
| Polymyxin B  +R^1^ | MIC (mg/L) | 64 + 16 | 2 + 256 | 1 + 32 | 0.06 + 512 | 32 + 64 | 32 + 64 | 512 + 32 |
|  | FICI^2^ | >0.5, <0.75 | >0.25, <0.5 | >0, <0.06 | >0.25, <0.5 | >0.5, <0.75 | >0.5, <0.75 | >0.75, <1 |
|  | Fold change in MIC decrease^2^ | >8 | 32 | >512 | 1024 | 8 | >16 | >1 |
|  | MBC (mg/L) | 256 + 16 | 32 + 256 | 1 + 32 | 64 + 512 | 64 + 64 | 64 + 64 | 512 + 32 |
| Colistin  +R^1^ | MIC (mg/L) | 64 + 16 | 2 + 512 | 1 + 32 | 0.5 + 512 | 32 + 64 | 32 + 64 | 256 + 32 |
|  | FICI^2^ | >0.5, <0.75 | >0.25, <0.5 | >0, <0.06 | >0.25, <0.5 | >0.5, <0.75 | >0.5, <0.75 | >0.75, <1 |
|  | Fold change in MIC decrease^2^ | >8 | 128 | >512 | >1024 | 16 | 16 | 2 |
|  | MBC (mg/L) | 256 + 16 | 64 + 256 | 1 + 32 | 128 + 512 | 128 + 64 | 64 + 64 | 512 + 32 |
| Polymyxin B  +EAR^1^ | MIC (mg/L) | 256 + 1 | 16 + 0.125 | <0.03 + 1 | 8 + 512 | 32 + 0.5 | 8 + 1 | 256 + 0.5 |
|  | FICI^2^ | >0.75, <1 | >0.25, <0.5 | >0, <0.06 | >0.5, <0.75 | >0.5, <0.75 | >0.5, <0.75 | >0.75, <1 |
|  | Fold change in MIC decrease^2^ | >2 | 4 | >16384 | 8 | 8 | >64 | >2 |
|  | MBC (mg/L) | 256 + 1 | 64 + 0.125 | 0.03 + 2 | 16 + 512 | 256 + 0.5 | 64 + 1 | 256 + 0.5 |
| Colistin  +EAR^1^ | MIC (mg/L) | 128 + 1 | 64 + 0.125 | <0.03 + 1 | 4 + 512 | 64 + 0.5 | 2 + 1 | 128 + 0.5 |
|  | FICI^2^ | >0.75, <1 | >0.25, <0.5 | >0, <0.06 | >0.5, <0.75 | >0.5, <0.75 | >0.5, <0.75 | >0.75, <1 |
|  | Fold change in MIC decrease^2^ | >4 | 4 | >16384 | >128 | 8 | 256 | 4 |
|  | MBC (mg/L) | 256 + 1 | 256 + 0.125 | 0.03 + 2 | 16 + 512 | 256 + 0.5 | 16 + 1 | 256 + 0.5 |
| Polymyxin B  +CE^1^ | MIC (mg/L) | 128 + 156 | 32 + 5000 | 128 + 2500 | 32 + 5000 | 128 + 156 | 256 + 312 | 128 + 312 |
|  | FICI^2^ | >0.75, <1 | >0.75, <1 | >0.5, <0.75 | >0.75, <1 | >0.75, <1 | >0.75, <1 | >0.75, <1 |
|  | Fold change in MIC decrease^2^ | >4 | 2 | >4 | 2 | 2 | >2 | >4 |
|  | MBC (mg/L) | 512 + 156 | 32 + 5000 | 512 + 2500 | 32 + 5000 | 256 + 156 | 512 + 625 | 512 + 625 |
| Colistin  +CE^1^ | MIC (mg/L) | 256 + 156 | 64 + 5000 | 128 + 2500 | 512 + 5000 | 256 + 156 | 256 + 312 | 128 + 312 |
|  | FICI^2^ | >0.75, <1 | >0.75, <1 | >0.5, <0.75 | >0.75, <1 | >0.75, <1 | >0.75, <1 | >0.75, <1 |
|  | Fold change in MIC decrease^2^ | >2 | 4 | >4 | >1 | 2 | 2 | 4 |
|  | MBC (mg/L) | 512 + 156 | 256 + 5000 | 512 + 2500 | 512 + 5000 | 512 + 156 | 512 + 312 | 512 + 312 |

E, emodin; AE, aloe emodin; R, rhein; EAR, emodin/ aloe emodin/ rhein mass ratios of 1:1:1; CE, crude extract of *A. barbadensis* by Lac-Glu solvent; PMB, polymyxin B; CST, colistin; FICI, fractional inhibitory concentration index; MIC, minimum inhibitory concentrations; MBC, minimum bactericidal concentration.

^1^ The values after the plus are optimal concentrations of E, AE, R, EAR, and CE.

^2^ These values are calculated based on the MIC values provided in Table 1.

**Supplementary Table 2.** The MIC (mg/L) values of PMB and CST against polymyxin-resistant *A. baumannii* 1415E in the presence of E, AE, R, EAR, or CE under different concentrations of EDTA.

| Compounds | Contents | Concentration of EDTA | | | |
| --- | --- | --- | --- | --- | --- |
|  |  | 64 | 128 | 256 | 512 |
| Polymyxin B  +E^1^ | MIC (mg/L) | >2 + 128 | >2 + 128 | <0.03 + 1 | <0.015 + 0.06 |
|  | Fold change in MIC decrease^2^ | <32 | <32 | >2048 | >4096 |
| Colistin  +E^1^ | MIC (mg/L) | 2 + 4 | 2 + 0.5 | <0.03 + 1 | <0.03 + 0.06 |
|  | Fold change in MIC decrease^2^ | >256 | >256 | >16384 | >16384 |
| Polymyxin B  +AE^1^ | MIC (mg/L) | >2 + 128 | 2 + 2 | <0.03 + 1 | <0.015 + 0.06 |
|  | Fold change in MIC decrease^2^ | <32 | 32 | >2048 | >4096 |
| Colistin  +AE^1^ | MIC (mg/L) | 2 + 4 | 2 + 1 | <0.03 + 1 | <0.03 + 0.06 |
|  | Fold change in MIC decrease^2^ | >256 | >128 | >16384 | >16384 |
| Polymyxin B  +R^1^ | MIC (mg/L) | 1 + 128 | 1 + 32 | <0.03 + 32 | <0.015 + 0.06 |
|  | Fold change in MIC decrease^2^ | 64 | 64 | >2048 | >4096 |
| Colistin  +R^1^ | MIC (mg/L) | 1 + 128 | 1 + 64 | <0.03 + 64 | <0.03 + 0.06 |
|  | Fold change in MIC decrease^2^ | >512 | >512 | >16384 | >16384 |
| Polymyxin B  +EAR^1^ | MIC (mg/L) | 2 + 32 | 0.5 + 128 | <0.03 + 4 | <0.015 + 0.06 |
|  | Fold change in MIC decrease^2^ | 32 | 128 | >2048 | >4096 |
| Colistin  +EAR^1^ | MIC (mg/L) | 2 + 16 | 1 + 16 | <0.03 + 8 | <0.03 + 0.06 |
|  | Fold change in MIC decrease^2^ | >256 | >512 | >16384 | >16384 |
| Polymyxin B  +CE^1^ | MIC (mg/L) | >2 + 5000 | >2 + 5000 | 1 + 5000 | <1 + 5000 |
|  | Fold change in MIC decrease^2^ | <32 | <32 | 64 | >64 |
| Colistin  +CE^1^ | MIC (mg/L) | >2 + 5000 | >2 + 5000 | 1 + 5000 | <1 + 5000 |
|  | Fold change in MIC decrease^2^ | <256 | <256 | >512 | >512 |

E, emodin; AE, aloe emodin; R, rhein; EAR, emodin/ aloe emodin/ rhein in a mass ratio of 1:1:1; CE, crude extract of *A. barbadensis* by Lac-Glu solvent; PMB, polymyxin B; CST, colistin; EDTA, ethylenediaminetetraacetic acid.

^1^ The values after the plus are optimal concentrations of E, AE, R, EAR, and CE.

^2^ These values are calculated based on the MIC values provided in Table 1.

**Supplementary Table 3.** The antibacterial and bactericidal activity of PMB and CST against polymyxin-resistant *A. baumannii* strains in the presence of E, AE, R, EAR, or CE supplemented with 256 mg/L EDTA.

| Compounds | Contents | 10213 | 07AC366 | 189B | 1415E | 6588E | 038E | 245B |
| --- | --- | --- | --- | --- | --- | --- | --- | --- |
| Polymyxin B  +E^1^ | MIC (mg/L) | 0.06 + 0.25 | 0.03 + 0.5 | <0.03 + 0.5 | <0.03 + 1 | 0.06 + 0.25 | 0.03 + 0.25 | 0.5 + 1 |
|  | FICI^2^ | >0.5, <0.75 | >0, <0.25 | >0, <0.25 | >0, <0.25 | >0.5, <0.75 | >0.5, <0.75 | >0.5, <0.75 |
|  | Fold change in MIC decrease^2^ | >8192 | 2048 | >16384 | >2048 | 4096 | >16384 | >1024 |
|  | MBC (mg/L) | 1 + 0.25 | 0.03 + 32 | 1 + 16 | 2 + 512 | 32 + 0.25 | 4 + 0.25 | 16 + 1 |
| Colistin  +E^1^ | MIC (mg/L) | 0.125 + 0.25 | 0.03 + 0.25 | <0.03 +0.25 | <0.03 + 1 | 0.06 + 0.25 | 0.06 + 0.25 | 0.5 + 1 |
|  | FICI^2^ | >0.5, <0.75 | >0, <0.25 | >0, <0.25 | >0, <0.25 | >0.5, <0.75 | >0.5, <0.75 | >0.5, <0.75 |
|  | Fold change in MIC decrease^2^ | >4096 | 8192 | >16384 | >16384 | 8192 | 8192 | 1024 |
|  | MBC (mg/L) | 2 + 0.25 | 0.03 + 16 | 1 + 1 | 1 + 512 | 32 + 0.25 | 4 + 0.25 | 16 + 1 |
| Polymyxin B  +AE^1^ | MIC (mg/L) | 0.125 + 1 | 0.03 + 0.5 | <0.03 + 1 | <0.03 + 1 | 0.06 + 1 | 0.06 + 2 | 0.5 + 1 |
|  | FICI^2^ | >0.5, <0.75 | >0, <0.25 | >0, <0.25 | >0, <0.25 | >0.5, <0.75 | >0.5, <0.75 | >0.5, <0.75 |
|  | Fold change in MIC decrease^2^ | >4096 | 2048 | >16384 | >2048 | 4096 | >8192 | >1024 |
|  | MBC (mg/L) | 1 + 1 | 0.06 + 0.5 | <0.03 + 2 | 2 + 512 | 8 + 1 | 2 + 2 | 16 + 1 |
| Colistin  +AE^1^ | MIC (mg/L) | 0.125 + 1 | 0.03 + 0.25 | <0.03 + 1 | <0.03 + 1 | 0.06 + 1 | 0.06 + 2 | 1 + 1 |
|  | FICI^2^ | >0.5, <0.75 | >0, <0.25 | >0, <0.25 | >0, <0.25 | >0.5, <0.75 | >0.5, <0.75 | >0.5, <0.75 |
|  | Fold change in MIC decrease^2^ | >4096 | 8192 | >16384 | >16384 | 8192 | 8192 | 512 |
|  | MBC (mg/L) | 2 + 1 | 0.06 + 0.25 | <0.03 + 2 | 1 + 512 | 8 + 1 | 2 + 2 | 16 + 1 |
| Polymyxin B  +R^1^ | MIC (mg/L) | 0.25 + 16 | 0.03 + 0.5 | <0.03 + 32 | <0.03 + 32 | 0.125 + 64 | 0.125 + 64 | 1 + 32 |
|  | FICI^2^ | >0.5, <0.75 | >0, <0.25 | >0, <0.25 | >0, <0.25 | >0.5, <0.75 | >0.5, <0.75 | >0.5, <0.75 |
|  | Fold change in MIC decrease^2^ | >2048 | 2048 | >16384 | >2048 | 2048 | >4096 | >512 |
|  | MBC (mg/L) | 2 + 16 | 0.03 + 128 | <0.03 + 32 | 0.03 + 512 | 16 + 64 | 2 + 64 | 8 + 32 |
| Colistin  +R^1^ | MIC (mg/L) | 0.125 + 16 | 0.03 + 0.25 | <0.03 + 16 | <0.03 + 64 | 0.125 + 64 | 0.06 + 64 | 1 + 32 |
|  | FICI^2^ | >0.5, <0.75 | >0, <0.25 | >0, <0.25 | >0, <0.25 | >0.5, <0.75 | >0.5, <0.75 | >0.5, <0.75 |
|  | Fold change in MIC decrease^2^ | >4096 | 8192 | >16384 | >16384 | 4096 | 8192 | 512 |
|  | MBC (mg/L) | 2 + 16 | 0.03 + 128 | <0.03 + 16 | 0.03 + 512 | 32 + 64 | 4 + 64 | 8 + 32 |
| Polymyxin B  +EAR^1^ | MIC (mg/L) | <0.06 + 0.5 | <0.03 + 0.125 | <0.03 + 0.5 | <0.03 + 4 | <0.03 + 0.5 | <0.06 + 0.5 | <1 + 0.25 |
|  | FICI^2^ | >0.25, <0.5 | >0, <0.25 | >0, <0.25 | >0, <0.25 | >0.25, <0.5 | >0.25, <0.5 | >0.25, <0.5 |
|  | Fold change in MIC decrease^2^ | >8192 | >2048 | >16384 | >2048 | >8192 | >8192 | >512 |
|  | MBC (mg/L) | <0.03 + 1 | 1 + 0.125 | <0.03 + 0.5 | 0.25 + 16 | 1 + 1 | 2 + 1 | 8 + 0.5 |
| Colistin  +EAR^1^ | MIC (mg/L) | <0.06 + 0.5 | <0.03 + 0.125 | <0.03 + 0.5 | <0.03 + 8 | <0.03 + 0.5 | <0.06 + 0.5 | <1 + 0.25 |
|  | FICI^2^ | >0.25, <0.5 | >0, <0.25 | >0, <0.25 | >0, <0.25 | >0.25, <0.5 | >0.25, <0.5 | >0.25, <0.5 |
|  | Fold change in MIC decrease^2^ | >8192 | >8192 | >16384 | >16384 | >16384 | >8192 | >512 |
|  | MBC (mg/L) | <0.03 + 1 | <0.03 + 0.125 | <0.03 + 0.5 | 0.25 + 16 | 2 + 1 | 2 + 1 | 16 + 0.5 |
| Polymyxin B  +CE^1^ | MIC (mg/L) | 32 + 156 | 1 + 5000 | 64 + 2500 | 1 + 5000 | 64 + 156 | 4 + 312 | 16 + 312 |
|  | FICI^2^ | >0.5, <0.75 | >0.5, <0.75 | >0.5, <0.75 | >0.5, <0.75 | >0.5, <0.75 | >0.5, <0.75 | >0.5, <0.75 |
|  | Fold change in MIC decrease^2^ | >16 | 64 | >8 | 64 | 4 | >128 | >32 |
|  | MBC (mg/L) | 32 + 156 | 1 + 5000 | 128 + 2500 | 1 + 5000 | 64 + 156 | 4 + 312 | 16 + 312 |
| Colistin  +CE^1^ | MIC (mg/L) | 32 + 156 | 1 + 5000 | 32 + 2500 | 1 + 5000 | 32 + 156 | 4 + 312 | 32 + 312 |
|  | FICI^2^ | >0.5, <0.75 | >0.5, <0.75 | >0.5, <0.75 | >0.5, <0.75 | >0.5, <0.75 | >0.5, <0.75 | >0.5, <0.75 |
|  | Fold change in MIC decrease^2^ | >16 | 256 | >16 | >512 | 16 | 128 | 16 |
|  | MBC (mg/L) | 32 + 156 | 1 + 5000 | 64 + 2500 | 1 + 5000 | 64 + 156 | 4 + 625 | 32 + 625 |
| Polymyxin B  +EDTA^1^ | MIC (mg/L) | 64 + 256 | 2 + 256 | 128 + 256 | 2 + 256 | 64 + 256 | 8 + 256 | 32 + 256 |
|  | FICI^2^ | >0.25, <0.5 | >0.25, <0.5 | >0.25, <0.5 | >0.25, <0.5 | 0.5 | >0.25, <0.5 | >0.25, <0.5 |
|  | Fold change in MIC decrease^2^ | >8 | 32 | >4 | 32 | 4 | >64 | >16 |
|  | MBC (mg/L) | 64 + 256 | 2 + 256 | 128 + 256 | 4 + 256 | 64 + 256 | 8 + 256 | 32 + 256 |
| Colistin  +EDTA^1^ | MIC (mg/L) | 64 + 256 | 1 + 256 | 128 + 256 | 2 + 256 | 64 + 256 | 8 + 256 | 64 + 256 |
|  | FICI^2^ | >0.25, <0.5 | >0.25, <0.5 | >0.25, <0.5 | >0.25, <0.5 | >0.25, <0.5 | >0.25, <0.5 | >0.25, <0.5 |
|  | Fold change in MIC decrease^2^ | >8 | 256 | >4 | >256 | 8 | 64 | 8 |
|  | MBC (mg/L) | 64 + 256 | 1 + 256 | 128 + 256 | 2 + 256 | 64 + 256 | 8 + 256 | 64 + 256 |
| EAR  +EDTA^1^ | MIC (mg/L) | 1 + 256 | 4 + 256 | 1 + 256 | 32 + 256 | 1 + 256 | 1 + 256 | 0.5 + 256 |
|  | FICI^2^ | >0.75, <1 | >1, <1.25 | >0.25, <0.5 | >0.25, <0.5 | >0.75, <1 | >0.5, <0.75 | >0.5, <0.75 |
|  | MBC (mg/L) | 16 + 256 | 1024 + 256 | 4 + 256 | 2048 + 256 | 2 + 256 | 32 + 256 | 32 + 256 |

E, emodin; AE, aloe emodin; R, rhein; EAR, emodin/ aloe emodin/ rhein in a mass ratio of 1:1:1; CE, crude extract of *A. barbadensis* by Lac-Glu solvent; PMB, polymyxin B; CST, colistin; FICI, fractional inhibitory concentration index; EDTA, ethylenediaminetetraacetic acid; MIC, minimum inhibitory concentrations; MBC, minimum bactericidal concentration.

^1^ The values after the plus are optimal concentrations of E, AE, R, EAR, and CE.

^2^ These values are calculated based on the MIC values provided in Table 1.
